# Hale’s Tours in Singapore and Hong Kong

**DOI:** 10.1080/17460654.2025.2501566

**Published:** 2025-06-18

**Authors:** Mario Slugan, Ata’ Hanifee, Weijia Zeng

**Affiliations:** Film, Queen Mary University of London, London, UK

## Abstract

Since Raymond Fielding’s essay on the subject, Hale’s Tours and Scenes of the World – an exciting multi-sensory simulation of train travel involving wagon-like auditorium and moving image projection – have received noteworthy attention from early cinema scholars. Despite Hale’s Tours being a worldwide phenomenon, however, the focus has been on North America and Western Europe. To our knowledge, there are only two properly referenced contributions on Hale’s Tours (or its clones) outside these regions – in Singapore and Rio de Janeiro. Focusing on regions outside North America and Western Europe, however, is crucial for arriving at a more representative understanding of historical spectatorship and the affects the device produced. By drawing on English, Chinese, and Malay sources, this essay, therefore, expands our understanding of the Hale’s Tours by investigating their first appearances in Asia in Singapore (1908) and Hong Kong (1912).

Following Raymond Fielding’s (1970) piece on the subject, Hale’s Tours and Scenes of the World have received considerable attention from early cinema scholars. Historians have discussed this early 20th century multi-sensory simulation of train travel involving train-like auditorium and moving image projection from various perspectives including its patent history, design, popularity, profits and losses, films shown, promotion strategies, and audience experiences. Concerning locales, we have learned about Hale’s Tours in the US (Fielding 1970, Musser 1990, Rabinovitz 2012), England (Hayes 2009), Norway (Iversen 2001), and Ireland (Zimmermann 2008). Yet, as many scholars repeatedly point out, Hale’s Tours were a worldwide phenomenon. According to Rabinovitz, for instance, “By the end of the 1906 summer season, Hale’s Tours have also spread to leading world capitals – there are records of installations in Mexico City, Havana, Melbourne, Paris, London, Berlin, Bremen, Hamburg, Hong Kong, and Johannesburg” (2012, 79).

Despite this, however, we are familiar with only two properly referenced multi-page accounts of the ride outside of Europe and North America.^1^ Nadi Tofighian (2013, 213-216) discusses Hale’s Tours in Singapore in 1908 focusing on the advertising campaign that surrounded it and the films that predominantly showed European and North American scenes. Within the context of desire for global travel in Brazil, Maite Conde (2018, 44-46) briefly addresses Hale’s Tours clones dubbed “Automobile Cinema” and “Global Railway” presented by a local businessman in Rio de Janeiro in 1905.^2^ If we are to understand the global appeal of the amusement ride to businesspeople and audiences alike, however, we should arguably investigate how the ride operated in other regions as well. Here, therefore, we provide a history of the attraction in Singapore and Hong Kong, two key regional hubs in Asia where Hale’s Tours arrived first. While this is primarily a historical paper unearthing fundamental information about Hale’s Tours in these cities, there are more theoretical concerns which motivate and can make use of this work. For instance, we would be well advised to understand the place Hale’s Tours had in what has been dubbed colonial modernity, i.e. the colonies-specific modernity resulting from the interaction of local forces and European colonialism. One way to think this through is to consider whether Hale’s Tours were foreign owned and operated or whether they offered opportunities for locals to enter the rapidly expanding film business. Another, as Tofighian demonstrates, is to appreciate that presenting films depicting views of predominantly North America and Europe as a part of Hale’s Tours would have had different functions depending on whether they were screened in imperial centres or colonial settlements and, in the latter case, whether they catered to colonial elites or local audiences.^3^

Speaking of audience experiences, focusing on the regions outside of Europe and North America is crucial for arriving at a more representative understanding of historical spectatorship. During the global heyday of Hale’s Tours between 1905 and 1910, more people lived in the colonies than they did in the imperial mother countries (Census of England and Wales 1911). The British Empire was only the most egregious case which at the time held sway over about a quarter of the world population but with the UK population amounting to only one tenth of the Empire. Moreover, while Hale’s Tours undoubtedly appeared in numerous big UK cities including London, Blackpool, Bristol, Leeds, Liverpool, Manchester, Nottingham, and amusement parks (Rabinovitz 2012; Hayes 2009), we should also not forget that were Hong Kong and Singapore in the UK, with more than 450 and 300 thousand inhabitants they would have been UK’s 5^th^ and 9^th^ largest city, respectively (Census of England and Wales 1911, 9; Fan 1974, 2; Saw 2012, 9).

Staying with audience experiences, identifying additional contemporary reports from around the globe can also help with deciding between competing understandings of the effects Hale’s Tours had based exclusively on the analyses of North American and European accounts. Since Fielding (1970), Hale’s Tours have been predominantly discussed in terms of cognitive illusion and false belief. Rabinovitz (2012) even went so far as to posit a ‘demented fellow’ as a model Hale’s Tours spectator. This ‘demented fellow’, according to a historical report from Vancouver, B.C (Thomas 1916) kept coming back to the show thinking there will be an actual crash. But there is an alternative. Slugan (2018, 2019), for instance, has downplayed Thomas’ account by emphasizing that it is not contemporary to Hale’s Tours and that it has been misquoted. Instead, based on contemporary reports from the US and UK press he has suggested that Hale’s Tours audiences were never naïve enough to falsely believe that they were taking a train ride. Rather, they imaginatively engaged with the simulation to play a game of make-believe in which they were traveling around the world.

Finally, it is not sufficient only to move the discussion of Hale’s Tours audiences (or those of any other film phenomena for that matter) from one region to another. It is also necessary to go beyond English print and use contemporary local language sources as well. Otherwise, we risk merely learning about the foreign colonialists’ engagement with the film phenomenon in question at best. And at worst we will end up with a skewed understanding of the locals’ engagement with it based on reports regularly inflected with supremacist and orientalist discourse.^4^ It is no secret that European accounts of colonial audiences faced with novel technologies regularly presented them as naïve locals stuck in magical thinking (cf. Bottomore 1999, appendix 2, 3; Burns 2000; Tofighian 2017). This opposition is, of course, not meant to erase a range of in-between social positions in the colonies and their relationship to race and language including the European poor, local elites (some of which would have been versed in English), and the relative statuses of different local ethnicities (Chinese in Singapore, for instance, were often business proprietors). But it is to emphasize that using only English (and European) primary sources will give us only a partial account of audience experiences.

In what follows, therefore, we draw on local archives, contemporary press, and local-language memoirs from Singapore and Hong Kong. Admittedly, contemporary English sources still dominate but this is because of the sheer volume of newspapers that existed in English and the privileged position the language enjoyed in the colonies at the time. For instance, both the incorporation files for Hale’s Tours in Singapore and the building plan of the theatre housing it are in English. But we do our best to supplement English-language sources with local-language ones, especially with key newspapers in Malay and Chinese. Given that in 1911 there were approximately 220 thousand Chinese and 42 thousand Malays in Singapore (Saw 2012, 29), we look at Chinese and Malay press alike. While the first Malay-language newspaper in Singapore was published in 1876 (Kuntom 1973), the first Chinese-language daily appeared in 1881 (Lee 2020). We consult *Utusan Malayu* which ran three days a week from 1907 to 1915 (later becoming a daily) and used Romanised script to appeal to potential Chinese readers (Kuntom 1973). Among Chinese newspapers we investigate *Lat Pau* (叻报, 1881–1932), *Union Times* (南洋总汇报, 1906–1948), and *Chong Shing Yit Pao* (中兴日报, 1907–1910) but find references to the ride only in *Lat Pau* and *Union Times*. In Hong Kong, where the first Chinese-language daily appeared at the end of 1850s (Scollon 1997), our main primary source is *Chinese Mail* (香港華字日報, 1872–1941), a crucial Chinese-language newspaper of the day, supplemented with later-day memoirs.

## Hale’s Tours at Beach Road, Singapore (and 165 Oxford Street, London)

Exhibited for the first time in Kansas City on 28 May 1905 (Musser 1990, 429) with the patent filed in March and approved by September (Slugan 2019), Hale’s Tours quickly spread through the US and around the globe. While there were many unauthorized copies like the aforementioned ‘Automobile Cinema’ and ‘Global Railway’, the patent-holders George C. Hale and Fred F. Gifford successfully sold numerous licenses and territorial rights for this early 4D technology.

By 1906 henry Iles had the rights for the ride in Britain, with the first one opening in London at 165 Oxford Street later that year (Hayes 2009). By the end of 1907, the company that was running the ride at this address, Hale’s Tours (Foreign Rights) Limited, sold the ‘right to use and deal with the device known as ‘Hales Tours’ in Singapore, Penang, The Federated Malay States and Java’ (Hale’s Tours Foreign Rights Eastern Company Limited 1908, 24) to Norman Dalrymple and James Guthrie MacTaggart for £8,000 or, adjusted for inflation, approximately £1,200,000 today.^5^ The two also obtained patent rights for the Straits Settlement and together with five more investors set up a company inventively called Hale’s Tours (Foreign Rights Eastern) Limited on 15 June 1908. The Company’s nominal capital was £5,500 divided into 5,500 £1 shares. 3,300 shares were given to Dalrymple and MacTaggart in lieu of their initial purchase of the rights while the remaining 2,200 shares could be purchased in an open subscription call from 23 June to 15 July 1908. Next to Dalrymple and MacTaggart, the Company’s directors were A. J. Ross, N. Reuben and Tan Kheam Hock, a local Chinese businessman and politician.

Hale’s Tours premiered for a private viewing on 29 July 1908 and next day for the paying public at Beach Road, ‘for the first time in Singapore and the Far East’.^6^ To our knowledge, this is the first appearance of the ride in Asia.

The press, English, Mandarin, and Malay alike, also provided a detailed explanation of how the attraction marshalled visual, sound, and ambulatory means for its immersive effect:

In a house by the sea on Beach Road, […] there is a train called ‘Pullman Car’ where there is no difference with real trains.^7^

The car holds 70 passengers each trip and as they sit there the particular journey chosen is unfolded before them […].^8^

There are two rows of seats in the train, and two seats in each row, with refreshments served. When the steam pipes start working, the lights in the train go off, and only the head of the train is bright.^9^

The principle on which Hales Tours are run is that the car is balanced and worked by a lever, giving the oscillation usually experienced in railway travelling. This, however, would not be sufficient illusion alone, unless a similar noise connected with railway travelling were produced. This is done by means of revolving wheels placed under the car, the noise being increased or decreased according to the nature of the track the train is supposed to be travelling, as represented by the moving picture on the screen. The sounds created when a train rattles over points, crosses bridges, or rumbles through a tunnel, and the whistle of the engine – a by no means unimportant item – is not forgotten. The real illusion is formed by the rapid approach of the rails on the picture towards the spectator. The whole effect is that you appear to be travelling in front of an engine at forty or fifty miles an hour.^10^

There we have it; there is no need to consult the patent papers for the potential audiences are privy to an explanation which addresses all the key aspects of the ride: the auditorium design in the form of a Pullman train car, the control of light within the auditorium, a system of pullers and levers which tilt and rock the makeshift car, a plethora of sound effects, and the projection of phantom rides or moving images that are (most often) taken from in front of the locomotive.

**Figure 1.**
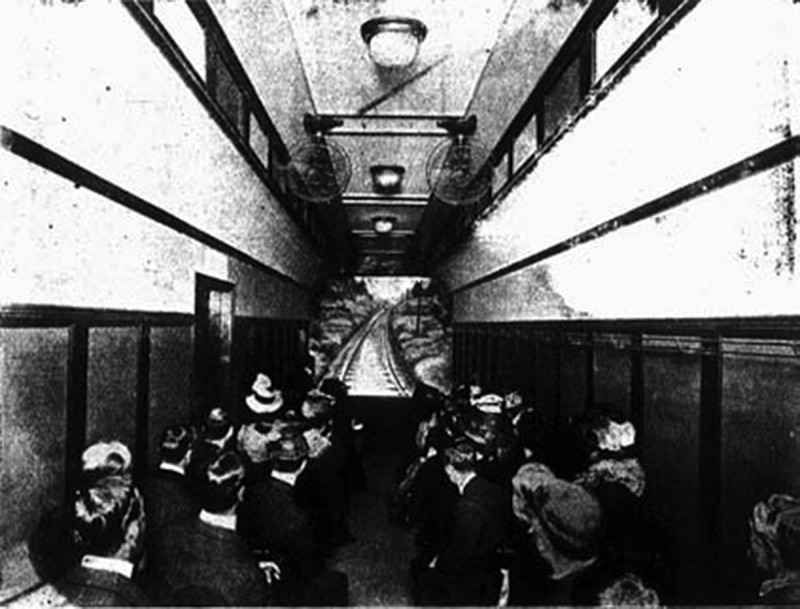
Inside Hale’s Tours. *Kinematograph and Lantern Weekly,* 1 October 1908, 481.

**Figure 2.**
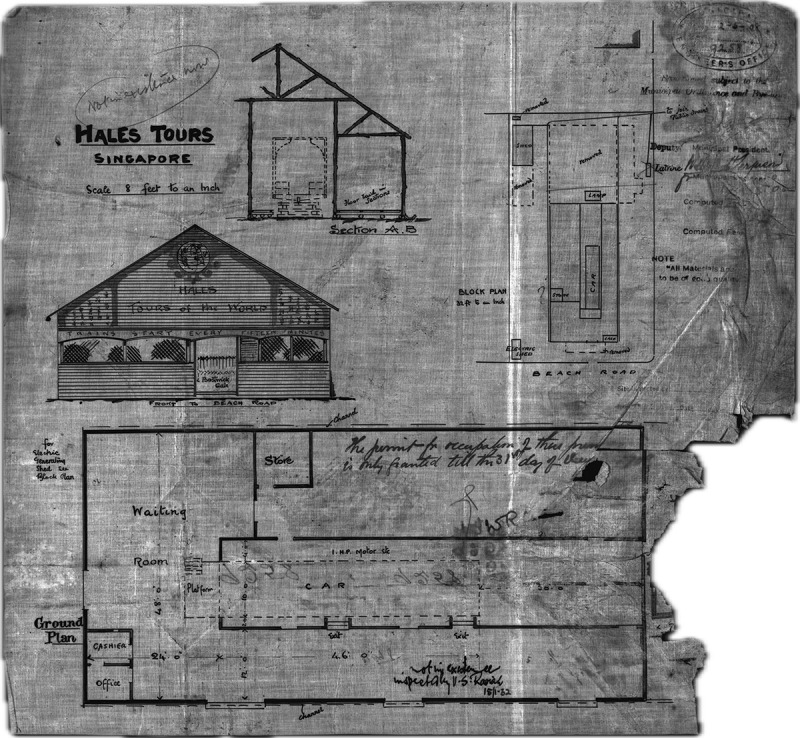
‘Hales Tours Building’, plan of building at Beach Road, 1908, reproduced with permission of National Archives of Singapore.^11^

The above description of the seating arrangement paired with the information from the Hale’s Tours (Foreign Rights Eastern) Limited Prospectus suggests that the car was of the same design as the one that appeared at 165 Oxford Street (minus the fans visible in the image) (Figure 1). Much like Hale’s and Gifford’s patent, the Prospectus speaks of two cars making a point that the second car will be installed later. However, the ads that have appeared while the show was in operation suggest that this never materialised. Moreover, we also know how the Beach Road House was developed to fit the new attraction (Figure 2).

The Prospectus also provides key information on the business logic behind the investment. Crucially, this is based on revenue figures from 165 Oxford Street provided to the Company by Hale’s Tours (Foreign Rights) Limited. In other words, looking at records kept in former colonies also enables us to give hard numbers when it comes to popularity and finances of the exhibition in the mother country. According to these figures, in 65 days from 6 October to 13 December 1907, there were 114,010 tickets sold at the cost of 6d (£3.8 today) for the box office taking of £2850 5s 0d or approximately £430,000 today.^12^ Based on this, the proprietors in Singapore estimated that if the attraction was open 25 days a month, 5 hours a day and if there were three rides per hour with 60 visitors charged at 20 cents per person, the revenue would amount to $4,500. After subtracting 10% royalty and $1,000 for various expenses (electricity, wages, rent, etc.), the monthly profits would be $3,050 or approximately £356.^13^ Even if they decreased the price to 10 cents, the Prospectus claims that the revenues would amount to $4,160 and profits to $2,744 or circa £320. Given the estimated capital expenditure costs for the installation of the first car of around £2,000, we can calculate that the Company expected to start turning profit in 6 months at the latest.^14^ Unfortunately for the proprietors, the paying exhibition lasted barely as long until it folded – from 30 July 1908 to 1 January 1909.^15^

But it certainly did not seem like that at the beginning. The first exhibition came to a great start with notable people in attendance. The opening was visited by the Governor of Singapore and the Chinese Council.^16^ In early September Siamese Royalty frequented the show.^17^ The reviews in Mandarin, Malay and English press alike were all glowing meaning that the exhibition was clearly open to all ethnicities.^18^ Special attention was given to the Chinese audiences who must have made up most of the visitors simply by virtue of being the majority ethnic group in Singapore (with Europeans making up less than 2% of the population) (Census of the Great Britain 1901). In November, the exhibition even closed for a day to commemorate the death of Emperess Dowager and her son Chinese Emperor.^19^ In a possible attempt to shore up Chinese patronage, the press claimed that the syndicate running the company was mostly owned by Chinese.^20^ It seems that, unlike the other cinema theatres at Beach Road and despite concerns raised in the papers, the proprietors even got electricity installations and the $670 bill for it covered by the Municipality which must have saved some money earmarked for capital expenses.^21^ The rides were also changed regularly to secure renewed interest. So, what happened? Why was the Company in liquidation by early January 1909 with all the holdings sold off by April 1909?

The biggest issue appears to have been a very poor business estimate. It is true that the Hale’s Tours in London folded around the same time due to a fall in audience interest and high long-term rent prices (Hayes 2009). But at the time the deal was made in mid-December 1907, the business in London was going strong so Singapore proprietors could not be blamed for not knowing what would happen next year in London. However, they should have still known better when it comes to Singapore i.e. that they could not reasonably expect to repeat the numbers from London even at the beginning of the show, given the differences in population sizes and cinema theatres per capita.

If we look at the London figures, there was on average of 1754 daily tickets sold. Owners for the Singapore rights have estimated 900 (5 × 3 × 60) spectators per day in the case 20 cents are charged. Interestingly, in the calculation for the lower 10 cents price they have estimated 1600 passengers by increasing the number of daily rides from 15 to 20 and the number of customers per ride to 80 (which clearly exceeds the ride capacity).^22^ So in the lower price estimate the number of daily tickets sold is almost the same as in London whereas in the higher price it is approximately half of it. But this does not take into consideration that with circa 4,5 million people London was about 15 times the size of Singapore. Even at a lower rate of 900 customers per day, in 11 months there would have been 300,000 tickets sold, one for every person in Singapore. This is simply not sustainable. If the proprietors thought this could somehow be offset by smaller competition in Singapore, London did not even have a higher density of cinemas and/or Hale’s Tours in London at the time either. By end of 1908 London had 4 Hale’s Tours (Hayes 2009) and 62 cinemas.^23^ With 1 Hale’s Tours and at least 5 cinemas at the time, Singapore had more moving image venues per capita than London.^24^

As another blow to their estimates, the pricing, wages and living costs would have favoured higher attendances in London as well. The actual cost of the ride in 165 Oxford Street London for the reported days was 6d, while in Singapore it started with 50 cents or 1s 2d (14d) - more than twice the London price. After three weeks second class tickets at 25 cents (7d) were introduced and by early December the prices fell to 20 cents (5.6d) for 1^st^ class (50 cents for 3 rides) and 10 cents (2.8d) for 2^nd^ class (20 cents for 3 rides).^25^ So the cheapest tickets were approximately half of the ticket price in London but these were only available for a month, while the standard ticket would have been even more expensive than the London one (7d as opposed to 6d). Moreover, average wages were also higher in the UK with workers there earning from about 2s d6 to 5s a day (Greasley 1989, 250) as opposed to their local counterparts in Singapore who received less than half as much i.e. somewhere between 50c (1s 2d) and $1 (2s 4d) (Tofighian 2013, 187–188). If we take prices of bread as a proxy for foodstuff expenses, then the prices in London and Singapore would have been approximately the same with 4lb bread at 5.8d (Sheppard and Norton 1957, 169; Tofighian 2013, 187). This means that while living expenses in the two cities were approximately the same and the salaries in London were twice that in Singapore, Hale’s Tours in Singapore were more expensive than in London both in relative and absolute terms.

While Hale’s Tours proved financially unviable in the mid-term in the US and the UK and in Singapore even in the short-term, for those who took the ride, it still afforded impressive experiences. The key selling point was certainly illusion of travel which was regularly advertised in the Singapore press: ‘The perfect illusion of rapid Railway travel amidst the loveliest scenery and most interesting places in the World’.^26^ But given their flair for exaggeration advertisements should take a back seat to audience reports when reconstructing historical experiences.

In fact, even when we move to other documents penned by proprietors but not aimed directly at advertising, as is the case with the legal documents for the incorporation of Hale’s Tours (Foreign Rights Eastern) Company Limited we see that the descriptions of the attraction are far more restrained:

The device known as ‘Hales Tours’ consists in the representation of a journey by rail or otherwise through any chosen part of the world. In the case of a journey by rail the passengers enter car built to represent a Pullman Car with the exception that the end which passengers face is removed and there the scenery of the country through which the train is supposed to be running is represented. This effect is produced by means of a biograph, the films of which have been prepared from actual photographs of the country represented. To complete the illusion of a journey the car has attached to it certain machinery which causes it to imitate the movements of a car forming part of a train travelling at high speed. The whole device combines to represent the effect of a journey by rail, the scenery appearing as the passenger [sic] would see it looking straight ahead from the front of the engine or rather as if her were seated in a car attached to the front of the engine.

(Hale’s Tours Foreign Rights Eastern Company Limited 1908, 24)

While even here there is an invocation of the term ‘illusion’, in the context of the opening and the closing sentences which emphasize the representational nature of the technology this is not a cognitive illusion of false belief but rather a much weaker illusion in the sense of dramatic illusion or make-believe. This much is confirmed by the invocation of conditionals (‘would see’) and ‘as if’ structures at the end.

The accounts of the rides in the press outside of obvious advertisements also speak of imaginative engagement as opposed to cognitive illusion, but admittedly on other occasions describe the ride in terms of false beliefs as well. In fact, the competing descriptions appear already in the first English-language July 30 reviews:

The first train […] made one imagine one’s-self back to the dear old London, Brighton and South Coast when the waiting passengers embarked. […] A curious illusion is created, and one can easily imagine one’s-self rounding curves on the railway tracks and dashing through tunnels and snow sheds to emerge upon mountain scenes in the Rockies.^27^

The Car is so constructed and devised that you have precisely the same sensation as of actual Railway travel. The oscillation, rapid movement, noise of travel, the sensation of rounding the curves as we approach them on the Railway track, the passing tunnels all tend to make the journey perfectly realistic and you leave the car with the full belief that you have actually travelled hundreds of miles in a most comfortable manner and visited the world’s best known and remarkable scenery leaving behind a recollection never to be effaced.^28^

While the first reviewer makes it clear that no false beliefs are formed but instead that the spectators actively imagine visiting different places and making turns on the tracks, the second commentator is as adamant in claiming that one is fooled into believing practically the very same things. The description of rides in terms of these distinct poles appear later in the English press as well.

For a brief spell one actually imagines himself transported in the old familiar English Railway or rushing through the tunnels and the ever-changing panorama of a Norway trip […] Advt.^29^

[I]t is an illusion of actual travel through the countries visited which it would be difficult to improve upon. One feels he is actually travelling at high speed through the various scenes.^30^

Crucially, given that the former quote is from an advertisement, a genre committed to hyperbole, and that it, contrary to the genre norms, emphasizes the restrained participatory imagination instead of the more dumbfounding false belief we are inclined to give more weight to the descriptions of historical experiences in terms of imaginative engagement. (In fact, the second quote could also be an advertisement merely masquerading as a news item.) This is not to deny that some people experienced false beliefs as reported by the clearly marked review above, but it is to suggest that it was less prevalent than imaginative participation that transformed the experience of the ride into a game of make-believe. In the press we can even find a suggestion that it is only those who lack imagination suffer false beliefs:

If you have no imagination, go to Hales Tours and experience realities. If you have a vivid imagination, go to Hales Tours and you will not be disappointed.^31^

For that commentator, the Chinese reviewer in *Lat Pau* would certainly have no imagination:

The crowd felt the train is vibrating while hearing the steam pipes like they were taking a ride. The first play is about the three different sceneries which they have been sightseeing. It is no different from personally being there, which makes the crowd confused and surprised. After the performance, one was just returning from a journey travelling at about forty or fifty miles per hour. The audience all praised that they never had similar experiences before.^32^

**Figure 3.**
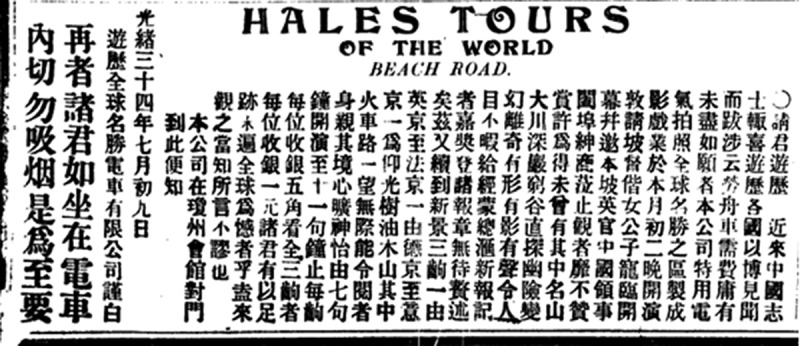
Advertisement for ‘Hales Tours’, *Union Times*, 12 August 1908, 3.

In other words, the report in Mandarin is adamant about the power of Hale’s Tours to generate false beliefs. The same point appears in a recurrent ad for the ride in Mandarin press (Figure 3): ‘The railways are endless, which makes the audience feel like just in front of the scenery relaxedly and joyfully’.^33^

**Figure 4.**
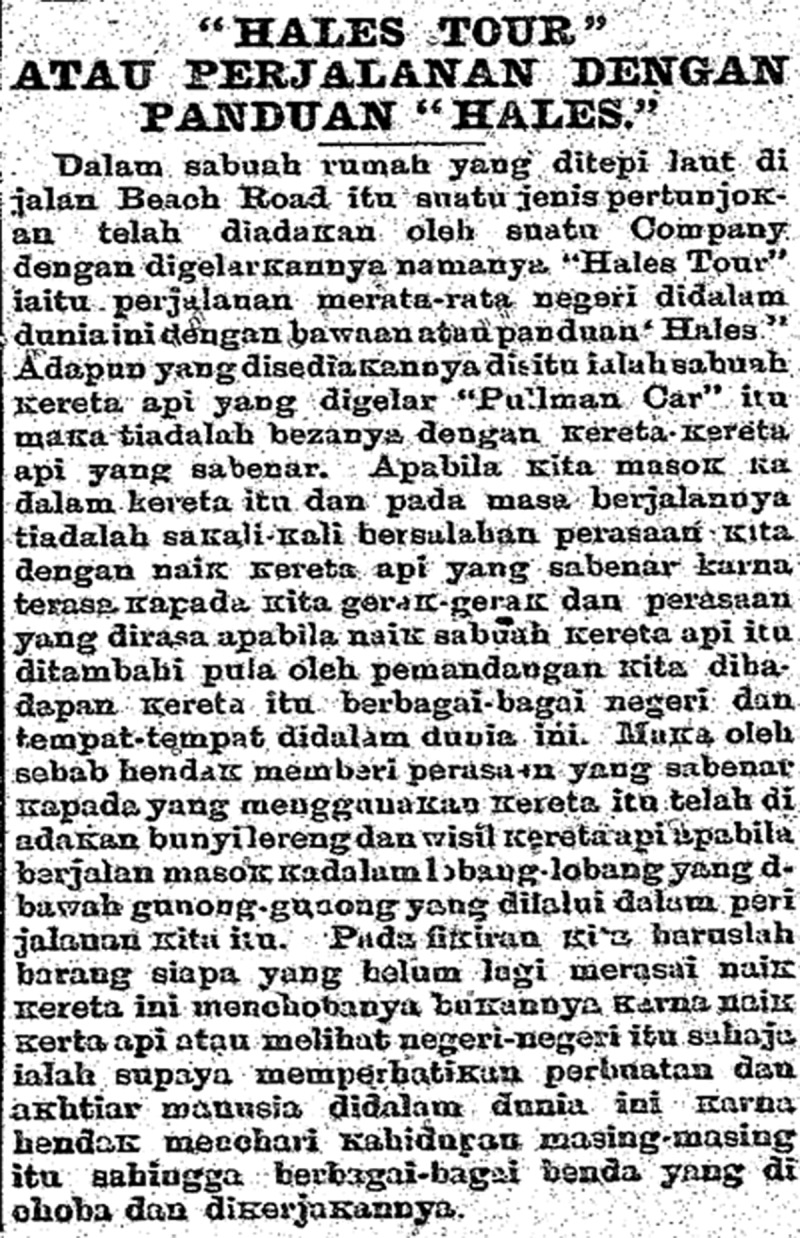
Report from *Utusan Melayu*, 11 August 1908, np.

While acknowledging the verisimilitude of visual, ambulatory, and sound effects, the review in Malay, however, moves away from imaginative engagement and illusory false belief as two poles for the description of the ride. Instead, it emphasizes the ride’s educational draw (Figure 4):

[T]here is a train called ‘Pullman Car’ where there is no difference with real trains. When we get into the train and at the time of its journey, there is no mistake in our feelings by riding a real train because we feel the movement and the feeling we feel when riding a train, plus the view we have in front of the train of various countries and places in this world. Because the train wants to give the real feeling to those who use the train, the sound of the train and the whistle of the train were made when walking into the tunnels under the mountains that we passed on our way. In our mind, anyone who has not yet experienced riding this train should try it, not because of riding the train or seeing the countries only, but to pay attention to the actions and efforts of people in this world because we want to discover each other’s [people abroad] lives and the various things they tried and worked on.^34^

The reviewer starts off with an account of how the auditorium is made to look visually undistinguishable from a train car, continues with how the attraction secures the bodily impression that one is moving while knowing quite well that they are not, and ends with a discussion of auditory effects contributing to the overall impression. Yet the main reason why, according to the reviewer, the ride is worth the visit is not sensory realism but learning about other people’s lives and practices.

And this brings us to the question of Eurocentrism raised by Tofighian. It is undeniable that the greatest bulk of films shown in Singapore depicted North America and Europe effectively implying that only these two regions make up the world in ‘Hales Tours of the World’.^35^ While statistics about film content do not lie, this does not necessarily mean that the Singapore audiences would have found that troublesome.

Obviously, for Europeans such film selection would be welcome as it would make them fondly reminisce about the mother country: ‘The first train […] made one imagine one’s-self back to the dear old London, Brighton and South Coast when the waiting passengers embarked’.^36^ In fact, here, for this effect it was sufficient for the ride to be shot in Europe as it was not even of the UK but of ‘A Trip to Norway’.^37^

But even for locals the dominance of films from North America and Europe would not necessarily pose a problem. As the Malay-language review suggests, it is simply the discovery of other people’s cultures and lives that is well worth the visit. Advertisements in Chinese press, similarly, speak of ‘Chinese aspirants [who] have preferred to travel around the world to broaden their horizons’ and of Hale’s Tours as an affordable alternative to doing so (Figure 4).^38^ Certainly, it might be argued that precisely because they were embedded in a Eurocentric worldview locals and local elites like the Malay-speaking reviewer would not have necessarily recognised that the shows further propagate this worldview. But denying the possibility that the viewers genuinely enjoyed these scenes also robs them of their agency. After all, if at the time the locals were capable of anticolonial struggle, they were also surely capable of enjoying one type of entertainment as opposed to another. In the end, in not naming any specific country, the review, unlike the nostalgic English one, does not give any reason to believe that scenes from one world region are to be preferred to scenes from another.^39^

## ‘Scenic Railway’ at the University of Hong Kong bazaar and Land Office, Queen’s Road

**Figure 5.**
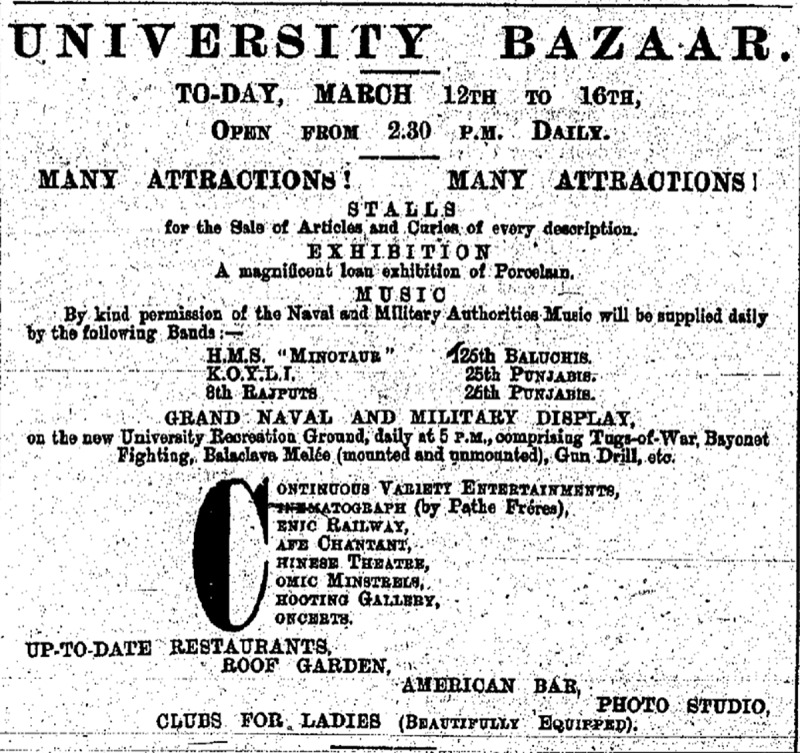
The university bazaar advertisement. *Hongkong Daily Press*, 12 March 1912, 3.

Although Rabinovitz (2001, 169; Rabinovitz 2004, 107; 2012, 77, 79) has repeatedly asserted that there were Hale’s Tours in Hong Kong by 1906, we have not been able to verify the claim.^40^ The earliest evidence of the amusement ride in Hong Kong that we have found dates to the opening of the University of Hong Kong and the accompanying fund-raising Bazaar that was originally planned for 11 to 16 March 1912 but was prolonged until 20 March (Figure 5).

The identification of the ride was further problematized by the fact that, unlike in Singapore where the attraction was referred to as ‘Hales Tours’ in Mandarin, Malay, and English press alike, in Hong Kong it was spoken about as ‘(s)cenic railway’ in English and as ‘幻遊火車’ in Chinese (which we translate as ‘Illusory Train Tour’).^41^ In English, at the time, ‘scenic railway’ usually referred to amusement rides like the one built for the Franco-British Exhibition in London in 1908 and covered in Hong Kong press.^42^ These, unlike Hale’s Tours, actually moved down the track and involved no projection or make-shift auditoriums. However, it is clear from an early account of the Bazaar and its entertainments that the commentator is talking about Hale’s Tours or at least one of its copies when referring to the scenic railway:

Yesterday afternoon, the representative of the ‘Post’, went over the building, on the kind invitation of Mr. C. Montague Ede, and saw what promises to be one of the most interesting features of the entertainments. This is the scenic railway, the first of its kind seen in Hongkong. The idea is certainly novel. In the strictest confidence he was told that the railway was the cheapest, the most efficient and speediest in the world. The car will seat fifty persons, and the ‘travellers’ will be taken round the island of Ceylon, up the Alps, from Brooklyn to New York, from Hongkong to Shaukiwan and up the peak all in the space of an hour. By an ingenious arrangement, the trippers will be able to experience peculiar rolling of a carriage when going round a dangerous curve, and also that shattering of the nerves which always accompanies what might have easily been a collision, averted only by the most expert driving.^43^

Admittedly, the description is not as detailed as the one in the Singapore press. For one, there is no revelation of what the technical nuts and bolts behind the ‘ingenious arrangement’ are. Rather the account remains on the level of effect – the swerve of inertia and the nail-biting thrill. In fact, despite the report on the University Bazaar opening which states that the ‘scenic railway […] has been described so often that a repetition would be superfluous’, we have not been able to find an account on par with the ones in Singapore press when it comes to revealing the ride’s technical details.^44^ There is not even a mention of motion pictures – either in the above lengthy citation from the *South Morning China Post* or in the advertisements and descriptions that would accompany the University Bazaar in the same newspapers as well as in *Hong Kong Daily Press*, *Hong Kong Telegraph*, *China Mail* or the Chinese-language *Chinese Mail*.^45^

Yet, we can be certain that these indeed were Hale’s Tours.^46^ The number of seats is clearly specified, the patrons are referred to as ‘travellers’, the ride is presented as the fastest and the most affordable, the bodily impression of movement is simulated, and the locales shift from Asia, through Europe to North America – something that would only be possible if the rides were virtual in the manner of Hale’s Tours. For those who need more convincing, once the attraction moved to another location, it was repeatedly advertised that ‘36 extra films’ have been purchased.^47^

**Figure 6.**
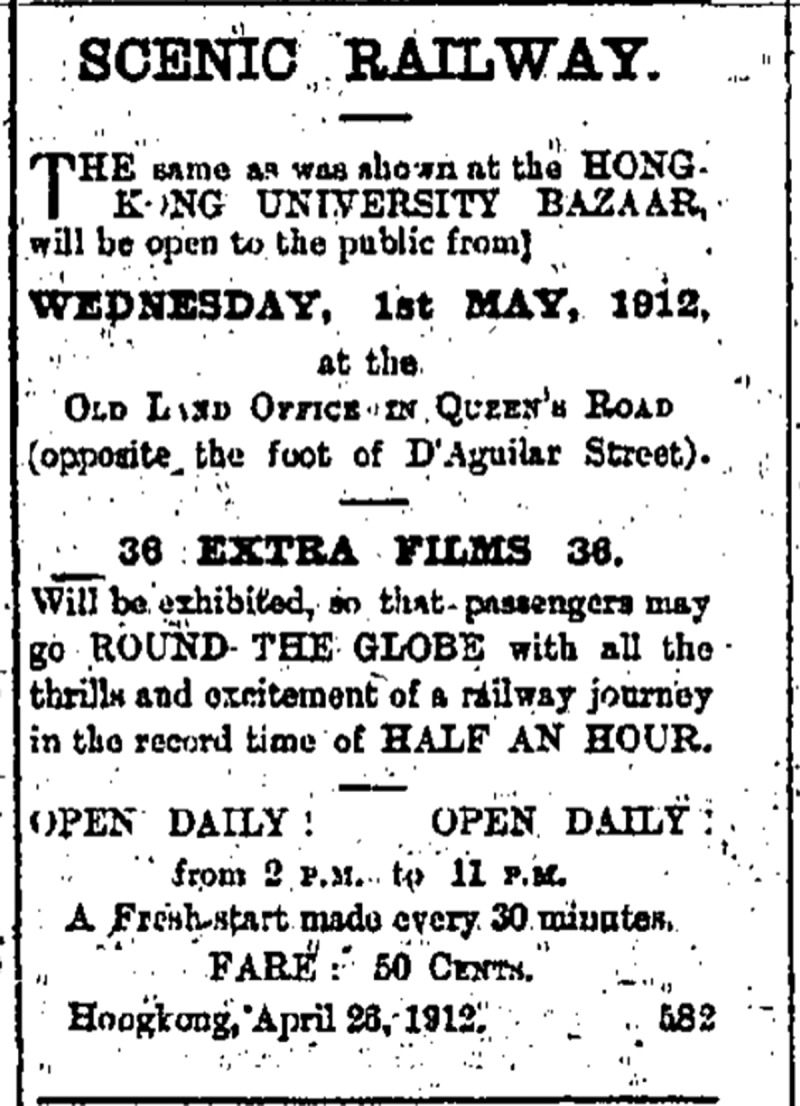
‘Scenic Railway’ advertisement at the old Land Office. *China Mail*, 27 April 1912, 3.

In its initial run, the Scenic Railway was in operation during the University Bazaar until 20 March.^48^ From altogether nearly $35,000 that the Bazaar made the Scenic Railway seems to have been the most popular attraction earning somewhat less than $9,000 (Other attractions included Pathé Frères Cinematograph, Café Chantant, Chinese theatre, comic minstrels, concerts, and shooting gallery).^49^ The Scenic Railway was auctioned the same month where it was sold for $10,000 to Mr. Pak Ming, somewhat disappointingly according to the press, because it was estimated it could gross up to $30,000 a year.^50^ As early as 26 April it was reported: ‘The old Land Office has been converted into a picture show. The scenic railway, which was such a success at the University Bazaar, has been installed with thirty-six new films and opens on 1st May’.^51^ Ads started appearing immediately thereafter (Figure 6).

As can be seen from the ad, the ride was open daily from 2 pm until 11 pm with a show every half an hour and 50 cents admission ticket which was reduced to 30 cents on 16 May.^52^ The attraction was in operation at least until 27 June according to the latest ad that we have been able to identify.^53^ Given that there was no notice similar to the Singapore announcement of a last show, it is probable that the ride continued for some time, running at least into August. A second-hand report about from a German-language missionary newsletter we have not been able to identify suggests that it was in operation as late as 16 August: ‘Sister Sidonie took her helpers to a cinematographic screening today. In rocking train cars, they watched parts of Norway, Denmark, Germany, etc. fly past’.^54^ In fact, another second-hand source – a Chinese-language *Hong Kong Cinema: The Silent Era, 1896–1929* – claims that the operations ended on 19 November 1912 and not for the lack of interest like in Singapore but because of the complaints about the noise the contraption was making (Yu 1996, 68).^55^

Whatever the exact final date was, the show must have lingered on at least in Chinese audiences’ memories, because the Hong Kong Theatre that later operated at that address advertised its location in Chinese as ‘the very place of 幻遊火車’. The ads on this theme appeared as early as 9 August 1915 and as late as 2 September 1916, the day after the theatre reopened as Hong Kong Cinematograph at the same location.^56^ In fact, as late as 1930 Wong Pui-kai reminisced on the ride giving, among other things, credence to the claim that the device was loud:

Many people have probably forgotten the ‘Illusory Train Tour’ in Hong Kong. This was the ‘Illusory Train Tour’ at the Hong Kong Theatre (now the site of Queen’s Theatre) more than ten years ago. The architectural form of this theatre was particularly different from those of other theatres at that time […]. Its construction structure is like a train (referring to the interior of the premise). There are machines on the ground and a floor board on top. The seats are arranged in two rows, like the second-class seats on the Kowloon-Canton Railway. When the movie screening commenced, the sound of the machine kept coming and the whole architecture was vibrating. There were also pictures shot when the train was moving. So during the screening, famous mountains, giant rivers, iron bridges and rivers are coming towards you. When you go through the tunnel, the whole premise is dark, and the machine is still vibrating. When you arrive at the station, the air horn or the bell rings and the commentator also shouts: ‘We have arrived at a certain place. Who wants to get off!? We are moving now, sit tight!’ Interspersed with the voices of people in the carriages, it is even more interesting. The screening time was divided into two shows, the first and last shows, in a secluded courtyard. The tickets were very cheap at that time, which made the audience get their money’s worth. (Wong 2017)^57^

We can find a similar comment about the noise from a memoir by Wong Yang Ching originally published in 1959 and also quoted by Yu:

Someone said that the [ride’s] discontinuation could not be attributed to unsatisfactory income, but because the loud noise of steam whistle and motor engine sound affected the neighborhood dreadfully. The local authority commanded the team to shift location out of consideration for peace. The proprietor could not find another place suitable for the performance, so the project came to an end.

(quoted in Yu 1996, 68; Wong 1959)

As we can glean from the advertisements and coverage, Hale’s Tours were frequented by Europeans and locals alike, both at the University Bazaar and the old Land Office. The attraction was initially purchased and operated by Europeans and then taken over by Chinese proprietors. It is unclear where Charles Montague Ede who set it up at the Bazaar originally acquired it from. Both the UK based Hale’s Tours (Foreign Rights) Limited and the Singapore-based Hale’s Tours (Eastern Foreign Rights) Limited were defunct by 1909. While it is not impossible that the device on sale in April 1909 in Singapore is the one that appeared in Hong Kong in March 1912 we must admit that we have no direct evidence to support such a claim.

Regardless of the provenance, we can compare the contemporary experiences of the ride in two cities and the diversity of views on offer. We have already noted that there appears to have been more appetite for the ride in Hong Kong than in Singapore and that, at the very least, it left a more lasting impression in the collective memory in the former. It is possible that the more geographically varied views from Asia, Europe, and North American and especially the images of Hong Kong on show already at the Bazaar, contributed to the popularity. The presence of a lecturer explaining the locations on view and revealed in commentary I discuss below might have also played a role.

Interestingly, despite the novelty of the attraction, unlike in the Singapore English press, there is no talk of illusion in its Hong Kong equivalent, either in ads or reviews, so this would not have been driving the ride’s appeal, at least not among the English speakers. The University Bazaar ads only mention the attraction by name and even the longer previews like the one from *The South China Morning Post* quoted above only stay at the level of bodily impressions.^58^ The reviews, similarly, make no appeal to illusion:

The Scenic Railway, with its novel and cheap trips to various parts of the world, is one of the most successful adjuncts to the bazaar, and it is chiefly due to Messrs. Ede, Ough, and Colonel Wrigly that the public is afforded the opportunity of participating in enjoyable and instructive trips into other lands whose characteristics are lucidly explained *en route*.^59^

[T]he scenic railway […] proved to be quite worth the entrance fee. There was a full cargo of passengers this trip. It was a wonderful train. First it brought us down from the Peak, then made an invisible jump to Shaukiwan, and next took us along Brooklyn Bridge after a marvellous non-stop run. But when we crashed through stout oaken buffers and found ourselves on the Continent, I felt like protesting. It seemed altogether too much for one dollar – at a bazaar, too! However, there was something compensating in the fact that the Continental place was not labelled. I heard it ascribed to no less than five different countries by people who had all ‘been there’. How great a thing is travel!^60^

The first review extols the edifying nature of the ride assisted by a lecturer. The second undeniably uses first-person vocabulary of travel to convey the experience but this is best described as imaginary travel rather than the one in which the passenger misunderstands their actual whereabouts. After all, given the commentator poking fun at other peoples’ certainty about where the European views were taken from and their alleged wide-reaching travels, the reviewer is too self-conscious a candidate for Rabinovitz’s ‘demented fellow’.

**Figure 7.**
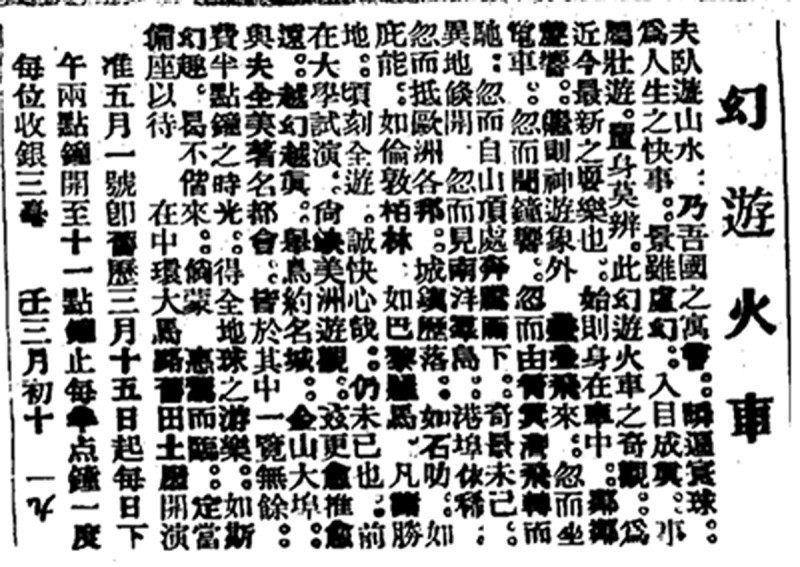
‘幻遊火車’ [‘Illusory Train Tour’] advertisement at the old Land Office. *Chinese Mail*, 12 June 1912, 6.

While we have not been able to identify contemporary reviews of the ride in Chinese press, there is a lengthy and recurrent ad that appears in *Chinese Mail* which we quote in full (Figure 7):

A man travels through mountains and rivers by lying in leisure – this is the allegory of our nation; swiftly traversing the globe is the joy of life. The scenery, though illusory, becomes reality when it comes into your eyes. The tour is grand and indistinguishable from reality. Such is the marvel of the Illusory Train Tour, the latest entertainment of recent times. Initially, one finds oneself inside the train, hearing the sounds of neighbouring villages. Soon after, the mind travels beyond the visible, with layers of scenes unfolding. Suddenly, one is sitting in an electric tram, hearing the ringing of bells; in an instant, soaring and speeding from Shaukiwan; the next moment, rushing down from a mountaintop. The strange scenes are unending, and distant lands unexpectedly appear. In a flash, one sees the islands of the Southeast Asia (Nanyang), with Hong Kong faintly visible; then, one arrives at the various countries of Europe, with towns laid out before the eyes, like Singapore, Penang, London, Berlin, Paris, and Rome. All the famous places are visited in an instant – truly a thrilling experience, and yet it continues. In a previous trial at the university, the spectacle lacked the sights of the Americas. Now, it has been pushed even further, with the more illusory becoming ever more real. The famous cities, like New York and San Francisco, as well as all the renowned metropolises of America, can all be seen in their entirety. In just half an hour, one enjoys the pleasure of traveling the entire globe. With such fantastic experiences, why not come along? Should you honour us with your presence, we will surely prepare a seat for you.

The ad appears to continuously waver between active imagination and passive illusion. It opens with a reference to the concept of *Woyou* (卧游) or spiritual traveling in bed developed by a Chinese landscape painter Zong Bing (Law 2011) to ground the experience of imaginary travel in the medium Chinese audiences would have been more familiar with. But in the next two sentences it insists that here there is an even stronger effect – a genuine illusion wherein one confuses representation for reality. Only a few sentences later, however, it is the mind that is knowingly imaginarily traveling again. Then, the passenger is sitting in a moving cart. Then again, the sights of America, while adding even more verisimilitude to the experience, never quite replace representation with reality. That even in the advertisement, a genre in which exaggeration is a feature rather than a bug, the proprietor never fully commits to illusion as the ride’s effect but rather vacillates between it and imagination, suggests that it is the latter that was a more typical response.

## Conclusion

This article has hopefully shown that if Hale’s Tours and Scenes of the World were a global phenomenon, then we should study it as such by expanding our primary sources both geographically and linguistically. And with any luck we have evinced the benefits of such an approach. Just to name a few, there is much to learn about the distribution networks between the center and the periphery, varied promotional strategies, differences in programing, exhibition contexts, pricing and admissions parameters, costs and financial projections, and nuances of historical experience across foreign and local audiences alike. And the colonial archive can even provide us with key information about the business of Hale’s Tours in mother country.

The historical data can also be taken further to test our theories. It can give us reason to refrain from blanket charges of Eurocentrism, at least when discussing audience preferences for the subjects depicted. There is no reason to a priori deny the possibility that the local audiences would have enjoyed a diet of North American and European sights any more than there is to deny the same for local views. Whereas the former could have had an educational appeal through virtual travel the latter could have brought the joy of seeing the familiar on screen.

The empirical findings can also provide additional information for our theories of spectatorship. While there were occasions where at least some audiences were fooled into believing that they were taking a ride through the locales depicted, most reports and even promotional material suggest that the ‘demented fellow’ is an unlikely candidate for a model spectator of Hale’s Tours. This is true of English and local-language reception alike. With Chinese-language reports being no more likely to cite cognitive illusion than English-language ones, this provides further evidence that locals were no more naïve than the colonizers. Furthermore, later reminiscences by Chinese writers, interestingly, do not mention cognitive illusion at all focusing rather on the noise the machinery produced. This is contrary to the tendency of the commentators reminiscing in the West (cf. Thomas 1916) to exaggerate the naivety of early audiences which went hand in hand with the rise of the myth of the panicking audience. In fact, there is one language in which cognitive illusion is never appealed to even in the contemporary description of the ride’s effect – Malay – suggesting that foreign elites were more credulous than at least some local audiences. Some reports even proposed a psychological explanation behind the engagement characterized by illusion when it does take place – the audience member’s lack of imagination.

Certainly, there is much more work to do. Further theorizing on the subject may relate this proposal about the lack of imagination to cultural factors such as familiarity with other local art forms and genres including landscape painting and shadow theatre. The matter of distribution networks within the periphery i.e. the question of where the attraction in Hong Kong comes from also remains. Another question is how widespread the Hale’s Tours in the (British) colonies were. Were Hale’s Tours opened in any of the locations that Dalrymple and McTaggart secured the rights for (Penang, The Federated Malay States or Java)? Johannesburg location cited by Rabinovitz is waiting to be confirmed. The sheer size of British India invites investigation for potential locales. Whatever the answers to these questions are, this article will have hopefully piqued an interest in them.

## Notes

1. Three, if we count a forthcoming paper by Slugan, Hanifee, and Zeng (2026).

2. Conde also discusses the ride elsewhere (Conde 2006, 2012) but information remains essentially the same.

3. In the rest of the paper, we use ‘local’ to refer to peoples living in the colonies who are of non-European descent even if they moved there from elsewhere. So, for instance, we consider Chinese in Singapore locals even if they immigrated there. We prefer ‘local’ to ‘non-European’ because the term is not a negation and because it hints at a different type of investment in the locale than that of the colonizers. Many migrants from China who moved to Singapore, for example, initially intended to return after earning enough money, but often ended up staying (Warren 2003).

4. We thank the anonymous reviewer for emphasizing this point.

5. £1 in 1907 is today worth about £150. https://www.officialdata.org/uk/inflation/1907?amount=1.

6. ‘Hale’s Tours’, *The Straits Times*, 27 July 1908, 1. https://eresources.nlb.gov.sg/newspapers/Digitised/Article/straitstimes19080727-1.2.2.2

7. ‘“Hales Tour” Atau Perjalanan Dengan Panduan “Hales”’, *Utusan Melayu*, 11 August 1908, np. All translations from Malay are by Ata Hanifee.

8. ‘Hales Tours’, *The Singapore Free Press and Mercantile Advertiser*, 29 July 1908, 5. https://eresources.nlb.gov.sg/newspapers/Digitised/Article/singfreepressb19080729-1.2.25.

9. ‘Novel & Fantastic Photoplay’ [‘影戲新奇’], *Lat Pau*, 30 July 1908, 8. All translations from Chinese are by Weijia Zeng.

10. ‘Private Opening Ceremony’, *The Singapore Free Press and Mercantile Advertiser*, 30 July 1908, 5. https://eresources.nlb.gov.sg/newspapers/Digitised/Article/singfreepressb19080730-1.2.26.

11. BCD; Building Control Division; 建筑管制组; Singapore. Public Works Department. Building Control Division. Accession Number: 9258/1908. Microfilm Number: 19990001016–0022. Source Reference Number: 9258/1908.

12. £1 = 20 s = 240 d.

13. ‘In 1905, the Straits dollar was fixed to the pound, at 2 shillings, 4 pence’ (Tofighian 2013: 59n219).

14. Here is a more detailed breakdown: car (£500), films and projector (£250), shipping and insurance (£50), travel expenses and salary for the expert installing the car (£150), car erection and electrical installations (£750), and advertising (£300).

15. ‘Hales Tours’, *The Straits Times*, 31 December 1908, 1. https://eresources.nlb.gov.sg/newspapers/Digitised/Article/straitstimes19081231-1.2.2.2

16. ‘Hales Tours’, *The Singapore Free Press and Mercantile Advertiser*, 30 July 1908, 5. https://eresources.nlb.gov.sg/newspapers/Digitised/Article/singfreepressb19080730-1.2.26. ‘Novel & Fantastic Photoplay’.

17. ‘Royalty at Hales Tours’, *The Straits Times*, 4 September 1908, 1. https://eresources.nlb.gov.sg/newspapers/Digitised/Article/straitstimes19080904-1.2.82.

18. ‘Novel & Fantastic Photoplay’; ‘“Hales Tour” Atau Perjalanan Dengan Panduan “Hales”’.

19. ‘Hales Tours’, *The Straits Times*, 19 November 1908, 1. https://eresources.nlb.gov.sg/newspapers/digitised/article/straitstimes19081119-1.2.2.3.

20. ‘Hale’s Tours’, *The Singapore Free Press and Mercantile Advertiser*, 30 July 1908, 5. https://eresources.nlb.gov.sg/newspapers/Digitised/Article/singfreepressb19080730-1.2.26. While it is possible that the shareholders were predominantly Chinese, it is certainly not the case that either the initial shareholders as founders or the board of Directors were.

21. ‘Supply for Electricity’, *The Straits Budget*, 23 July 1908, 1. https://eresources.nlb.gov.sg/newspapers/Digitised/Article/straitsbudget19080723-1.2.3. ‘Municipal Administration and Electricity’, *The Straits Budget*, 30 July 1908, 14. https://eresources.nlb.gov.sg/newspapers/digitised/article/straitsbudget19080730-1.2.56.8. ‘Hales Tours and Electricity’, *The Straits Budget*, 30 July 1908, 2. https://eresources.nlb.gov.sg/newspapers/digitised/article/straitsbudget19080730-1.2.6.

22. They have also increased the of days in a month from 25 to 26.

23. https://www.map.londonssilentcinemas.com/dhp-projects/londons-silent-cinemas/.

24. ‘Day by Day’, *The Straits Times*, 12 December 1908, 5. https://eresources.nlb.gov.sg/newspapers/digitised/article/straitstimes19081212-1.2.16.1.

25. ‘Hales Tours’, *The Straits Times*, 19 August 1908, 1. https://eresources.nlb.gov.sg/newspapers/digitised/article/straitstimes19080819-1.2.2.1. ‘Hales Tours’’, *The Singapore Free Press and Mercantile Advertiser*, 5 December 1908, 1. https://eresources.nlb.gov.sg/newspapers/digitised/article/singfreepressb19081205-1.2.3.3.

26. ‘Hales Tours’, *The Straits Times*, 21 September 1908, 1. https://eresources.nlb.gov.sg/newspapers/digitised/article/straitstimes19080921-1.2.2.1; ‘Hales Tours’, *The Singapore Free Press and Mercantile Advertiser*, 30 September 1908, 1. https://eresources.nlb.gov.sg/newspapers/digitised/article/singfreepressb19080930-1.2.4.1.

27. ‘Opening a Novel Entertainment at Beach Road’, The Straits Times, 30 July 1908, 8. https://eresources.nlb.gov.sg/newspapers/Digitised/Article/straitstimes19080730-1.2.92.

28. ‘Private Opening Ceremony’.

29. ‘Hales Tours’, *The Straits Times*, 12 October 1908, 8. https://eresources.nlb.gov.sg/newspapers/Digitised/Article/straitstimes19081012-1.2.82.

30. ‘Hale’s World Tours’, *The Straits Times*, 27 August 1908, 8. https://eresources.nlb.gov.sg/newspapers/Digitised/Article/straitstimes19080827-1.2.95.

31. *The Straits Times*, 9 September 1908, 6. https://eresources.nlb.gov.sg/newspapers/Digitised/Article/straitstimes19080909-1.2.36.

32. ‘Novel & Fantastic Photoplay’.

33. *Union Times*, ‘Hales Tours of the World’, 12 August 1908, 3; *Union Times*, ‘Hales Tours of the World’, 20 August 1908, 6; *Union Times*, ‘Hales Tours of the World’, 21 September 1908, 6.

34. ‘“Hales Tour” Atau Perjalanan Dengan Panduan “Hales”’.

35. ‘Hales Tours’, *The Straits Times*, 21 September 1908, 1.

36. ‘Opening a Novel Entertainment at Beach Road’.

37. Ibid.

38. *Union Times*, ‘Hales Tours of the World’, 12 August 1908, 3; *Union Times*, ‘Hales Tours of the World’, 20 August 1908, 6; *Union Times*, ‘Hales Tours of the World’, 21 September 1908, 6.

39. Given the aforementioned demographic and economic factors, it is unlikely that the selection of films which was biased towards North America and Europe had a significant impact on attendance.

40. Rabinovitz refers to the *Billboard*, 3 February 1906, 20 in making this claim but the source contains no information on this matter.

41. We would like to thank Jessica Siu-Yin Yeung for drawing our attention to this.

42. *South China Morning Post*, 28 May 1908, 11.

43. ‘The University Bazaar’, *South China Morning Post*, 7 March 1912, 6.

44. *Hong Kong Daily Press*, 12 March 1912, 5.

45. Cf. *Hong Kong Daily Press*, 12 March 1912, 4; *Hong Kong Telegraph*, 12 March 1912, 2; *China Mail*, 12 March 1912, 4, 5; *Chinese Mail*, 13 March 1912, 4.

46. Henceforth, when we write of Hale’s Tours in Hong Kong we mean Hale’s Tours or its copies.

47. *South China Morning Post*, 26 April 1912, 5.

48. ‘The University Bazaar’, *South China Morning Post*, 19 March 1912, 6.

49. All figures in this section are Hong Kong Dollars. ‘Hongkong University Bazaar’, *South China Morning Post*, 17 June 1912, 7. The press has followed the Bazaar’s takings from day 1 and reported that they fell considerably below the expected $100,000. Cf. ‘The Bazaar’, *South China Morning Post*, 13 March 1912, 7.

50. ‘Scenic Railway Sold’, *South China Morning Post*, 25 March 1912, 6; *China Mail*, 20 March 1912, 6.

51. *South China Morning Post*, 26 April 1912, 5; *South China Morning Post*, 29 April 1912, 2.

52. *South China Morning Post*, 16 May 1912, 5.

53. ‘幻遊火車’ [‘Illusory Train Tour’], *China Mail*, 27 June 1912, 4. The last ad in the English press that we found was in *Hongkong Telegraph*, 7 June 1912. The difference between English and Chinese sources is that while in English the daily ads simply stop on 7 June, it is unclear when the (presumably) daily ads stop because there is a gap in the *Chinese Mail* coverage at the Hong Kong Public Library that we consulted from 28 June 1912 to 19 February 1913.

54. https://gwulo.com/media/28098. My translation.

55. Given that Yu misidentifies the starting date as 15 May 1908 (instead of 1 May), the end date might also be inaccurate.

56. *Chinese Mail*, 9 August 1915, 3; *Chinese Mail*, 2 September 1916, 3; ‘The New Hongkong Cinematograph’, *The Hongkong Telegraph*, 30 August 1916, 8.

57. Also quoted in https://crumbtracker.blogspot.com/2017/11/phantom-ride.html.

58. Cf. ‘The University Bazaar’, *The Hongkong Telegraph*, 7 March 1912, 4. The 6 March preview did not actually include a ride because the required electric cable was disconnected for preparatory work

59. *China Mail*, 12 March 1912, 6.

60. ‘University Bazaar’, *South China Morning Post*, 12 March 1912, 3.
